# Supplementary material for: Analysis of PPARγ Signaling Activity in Psoriasis
Source: Int J Mol Sci. 2021 Aug 10;22(16):8603. doi: 10.3390/ijms22168603 (PMC8395241; doi:10.3390/ijms22168603)
Supplement: Supplementary file 1 [file ijms-22-08603-s001.zip › Supplemental materials_Analysis of PPARg signaling activity in psoriasis/Pathway models/Models images and html files/Model of PPARG signaling in psoriasis/2559.html]

CCR1


# Protein CCR1

|  |  |
| --- | --- |
| URN | urn:agi-llid:1230 |
| Total Entities | 0 |
| Connectivity | 3118 |
| Name | CCR1 |
| Description | C-C motif chemokine receptor 1 |
| Notes | This gene encodes a member of the beta chemokine receptor family, which is predicted to be a seven transmembrane protein similar to G protein-coupled receptors. The ligands of this receptor include macrophage inflammatory protein 1 alpha (MIP-1 alpha), regulated on activation normal T expressed and secreted protein (RANTES), monocyte chemoattractant protein 3 (MCP-3), and myeloid progenitor inhibitory factor-1 (MPIF-1). Chemokines and their receptors mediated signal transduction are critical for the recruitment of effector immune cells to the site of inflammation. Knockout studies of the mouse homolog suggested the roles of this gene in host protection from inflammatory response, and susceptibility to virus and parasite. This gene and other chemokine receptor genes, including CCR2, CCRL2, CCR3, CCR5 and CCXCR1, are found to form a gene cluster on chromosome 3p. [provided by RefSeq, Jul 2008] |
| Primary Cell Localization | Plasma membrane |
| Class | Receptor |

---

|  |  |
| --- | --- |
| Pathway | CC Chemokine Receptor Signaling |
|  | Mast-Cells De Novo Synthesized Mediators via IgE Independent Signaling |
|  | Nociception Expression Targets Signaling |
|  | CCR1 Expression Targets |
|  | Chemokines Signaling in Atherosclerosis |
|  | Proteins Involved in Myocarditis |
|  | Osteoclast Activation in Multiple Myeloma |
|  | Proteins Involved in Rheumatoid Arthritis |
|  | Proteins Involved in Endometriosis |
|  | Proteins Involved in Glomerulonephritis |
|  | CCL3 Expression Targets |
|  | CCL3L1 Expression Target |
|  | CCL16 Expression Targets |
|  | CCL5 Expression Targets |
|  | CCL7 Expression Target |
|  | CCL7 Expression Targets |
|  | CCL8 Expression Targets |
|  | CCL2 Expression Targets |
|  | TNF Receptors -> Expression Targets in Lymphoid System and Blood |
|  | C5AR1 -> Expression Targets in Lymphoid System and Blood |
|  | Model of PPARG signaling in psoriasis |
|  | PPARG negative regulators and targets |
|  | drug-target pairs direct regulation |

---

|  |  |
| --- | --- |
| Group | Chemokines, Growth Factors and Receptors Involved in Cancer Cells Chemotaxis |

---

|  |  |
| --- | --- |
| MedScan ID | 1230 |

---

|  |  |
| --- | --- |
| LocusLink ID | 1230 |
|  | 12768 |
|  | 57301 |

---

|  |  |
| --- | --- |
| Alias | CKR1\_HUMAN |
|  | Cmkbr1l I |
|  | SCYAR1 |
|  | CCR1 receptor |
|  | CMKBR1 |
|  | CCR1 |
|  | RANTES-R |
|  | chemokine (C-C) receptor I |
|  | chemokine (C-C) receptor 1,-like |
|  | CCR1 receptors |
|  | CMKR I |
|  | LD78 receptor |
|  | RANTES receptors |
|  | CMKBR I |
|  | chemokine receptor CCR1 |
|  | Ccr1l I |
|  | CC-chemokine receptor-1 |
|  | C-C chemokine receptor 1 |
|  | C-C chemokine receptor I |
|  | Ccr I |
|  | LD78 receptors |
|  | hCCR1 |
|  | antigen CD191 |
|  | chemokine receptors CCR1 |
|  | LOC57301 |
|  | chemokine (C-C) receptor 1 |
|  | CMKR1 |
|  | RANTES receptor |
|  | CKR-1 |
|  | SCYAR I |
|  | CKR I |
|  | chemokine (C-C motif) receptor 1 |
|  | MIP-1 alphaRL I |
|  | CC-CKR I |
|  | HM145 |
|  | CD191 antigen |
|  | chemokine (C-C motif) receptor I |
|  | chemokine (C-C motif) receptor 1-like 1 |
|  | CD191 |
|  | chemokine (C-C) receptor 1-like 1 |
|  | C-C motif chemokine receptor 1 |
|  | CC-CKR-1 |
|  | CKR1 |
|  | MIP1aR |
|  | C-C CKR-1 |
|  | CCR-1 |
|  | MIP-1alpha-R |
|  | macrophage inflammatory protein 1-alpha receptor |
|  | C-C chemokine receptor type 1 |
|  | Mip-1a-R |
|  | MIP-1 alpha R |
|  | MIP-1 alphaR |
|  | macrophage inflammatory protein-1 alpha receptor |

---

|  |  |
| --- | --- |
| GO ID | 0019957 |
|  | 0016493 |
|  | 0071791 |
|  | 0035717 |
|  | 0019956 |
|  | 0004950 |
|  | 0004435 |
|  | 0007186 |
|  | 0007187 |
|  | 0006816 |
|  | 0019722 |
|  | 0007155 |
|  | 0060326 |
|  | 0007166 |
|  | 0007267 |
|  | 0006874 |
|  | 0070098 |
|  | 0006935 |
|  | 0019221 |
|  | 0002407 |
|  | 0006887 |
|  | 0006955 |
|  | 0006954 |
|  | 0030502 |
|  | 0010629 |
|  | 0070374 |
|  | 0051928 |
|  | 0030335 |
|  | 0007204 |
|  | 0090026 |
|  | 0045672 |
|  | 0009611 |
|  | 0005737 |
|  | 0009897 |
|  | 0005887 |
|  | 0005886 |
|  | 0004930 |
|  | 0030595 |
|  | 0030099 |
|  | 0045824 |
|  | 0007165 |
|  | 0016021 |
|  | 0016020 |
|  | 0043025 |
|  | 0021549 |

---

|  |  |
| --- | --- |
| KEGG ID | hsa:1230 |
|  | mmu:12768 |

---

|  |  |
| --- | --- |
| Organism | Homo sapiens {Organism urn:agi-taxid:9606} |
|  | Mus musculus {Organism urn:agi-taxid:10090} |
|  | Rattus norvegicus {Organism urn:agi-taxid:10116} |

---

|  |  |
| --- | --- |
| Mouse chromosome position | 9 75.05 cM |

---

|  |  |
| --- | --- |
| OMIM ID | 601159 |

---

|  |  |
| --- | --- |
| Rat chromosome position | 8q32 |

---

|  |  |
| --- | --- |
| Hugo ID | 1602 |

---

|  |  |
| --- | --- |
| Human chromosome position | 3p21.31 |

---

|  |  |
| --- | --- |
| Swiss-Prot Accession | P32246 |
|  | Q5U003 |
|  | P32246.1 |
|  | P51675 |
|  | Q8BMH9 |
|  | Q8BVW4 |
|  | P51675.2 |
|  | Q9JLY8 |
|  | Q86VA9 |
|  | Q6ZWR7 |
|  | Q91VP9 |

---

|  |  |
| --- | --- |
| PIR ID | A45177 |
|  | I49339 |

---

|  |  |
| --- | --- |
| GenBank ID | NC\_000003 |
|  | NM\_001295 |
|  | NP\_001286 |
|  | AC104439 |
|  | CH471055 |
|  | EAW64756 |
|  | EAW64757 |
|  | AI089760 |
|  | BC051306 |
|  | AAH51306 |
|  | BC064991 |
|  | AAH64991 |
|  | BT019908 |
|  | AAV38711 |
|  | BT019909 |
|  | AAV38712 |
|  | BX337412 |
|  | CK903574 |
|  | D10925 |
|  | BAA01723 |
|  | L09230 |
|  | AAA58408 |
|  | L10918 |
|  | AAA36543 |
|  | P32246 |
|  | NC\_000075 |
|  | NM\_009912 |
|  | NP\_034042 |
|  | AC140491 |
|  | AC192862 |
|  | CH466671 |
|  | EDL37173 |
|  | U28404 |
|  | AAA89153 |
|  | AK031109 |
|  | BAC27256 |
|  | AK036597 |
|  | BAC29500 |
|  | AK036690 |
|  | BAC29535 |
|  | AK076275 |
|  | BAC36284 |
|  | BC011092 |
|  | AAH11092 |
|  | CT010178 |
|  | CAJ18386 |
|  | U29678 |
|  | AAA86119 |
|  | P51675 |
|  | NC\_005107 |
|  | NM\_020542 |
|  | NP\_065417 |
|  | AC\_000076 |
|  | AABR07071821 |
|  | AAHX01057714 |
|  | AF119381 |
|  | AAF34340 |
|  | CH473954 |
|  | EDL76748 |
|  | BC079207 |
|  | AAH79207 |

---

|  |  |
| --- | --- |
| Swiss-Prot ID | CCR1\_HUMAN |
|  | CCR1\_MOUSE |

---

|  |  |
| --- | --- |
| Cell Localization | Cell membrane |

---

|  |  |
| --- | --- |
| Ensembl ID | ENSG00000163823 |
|  | ENSP00000296140.3 |
|  | ENST00000296140.4 |
|  | ENSMUSG00000025804 |
|  | ENSMUSP00000026911.4 |
|  | ENSMUST00000026911.5 |
|  | ENSRNOG00000006715 |
|  | ENSRNOP00000008772.3 |
|  | ENSRNOT00000008772.4 |

---

|  |  |
| --- | --- |
| MGI ID | MGI:104618 |

---

|  |  |
| --- | --- |
| RGD ID | 708446 |

---
